# The Readiness Potential Reflects the Reliability of Action Consequence

**DOI:** 10.1038/s41598-018-30410-z

**Published:** 2018-08-08

**Authors:** Wen Wen, Rin Minohara, Shunsuke Hamasaki, Takaki Maeda, Qi An, Yusuke Tamura, Hiroshi Yamakawa, Atsushi Yamashita, Hajime Asama

**Affiliations:** 1 Department of Precision Engineering, the University of Tokyo, 7-3-1 Hongo, Bunkyo-ku, Tokyo, 113-8656 Japan; 2 Department of Neuropsychiatry, Keio University School of Medicine, 35 Shinanomachi, Shinjuku-ku, Tokyo, 160-8582 Japan; 3 Japan Society for the Promotion of Science, 5-3-1 Kojimachi, Chiyoda-ku, Tokyo, 102-0083 Japan; 4 Institute of Cognitive Neuroscience, University College London, Alexandra House, 17 Queen Square, London, WC1N 3AZ UK

## Abstract

Humans are capable of associating actions with their respective consequences if there is reliable contingency between them. The present study examined the link between the reliability of action consequence and the readiness potential (RP), which is a negative potential observed from about 1–2 s prior to the onset of an action with electroencephalography. In a condition of constant outcome, the participants’ voluntary action always triggered beep sounds; thus, they were able to perceive the contingency between their action and the sound. In contrast, in a condition of inconstant outcome, the participants’ actions only triggered the sound in half the trials. We found that both the early and late RPs were larger in the condition of constant compared to the condition of inconstant outcome. Our results showed that the RPs preceding the voluntary action reflected the reliability of action consequence. In other words, the action-effect contingency enhanced neural activities prior to the action.

## Introduction

Humans are capable of associating their actions with outcomes if there is contingency between them. Specifically, if an event always occurs immediately after an action and does not occur if the action is not performed, people may soon notice the association between the action and event. Developmental research on infancy showed that infants at very early ages (9–12 weeks) could perceive such action-effect contingency^1^. This indicates that the perception of contingency may be a very fundamental cognitive ability for humans. A previous study showed that positive action feedback increased action selection and suggested that action feedback may be rewarding to the organism^2^. However, it remains unknown how the association of an action with its consequence influences the neural processes preceding that action.

The present study examined the neurophysiological activity preceding the action using electroencephalography (EEG). We used the readiness potential (RP) as an index. The RP refers to a slow negative EEG potential that begins 1–2 s prior to a voluntary action^3,4^. It could only be observed by averaging multiple trials time-locked to the onset of the action. Prior research has linked the RP with free will or the intention to act^5–8^. The RP could also be influenced by several factors involved in the action, such as effort^9^, precision^10^, complexity^11^, and reward^12^. In the present study, we hypothesized that acquiring a reliable controllability over external events could be internally rewarding for the action, and, therefore, enhance the RP. To test this hypothesis, we examined the RP in a condition in which participants received constant outcomes of their actions and, thus, were able to reliably associate their action with the outcomes, compared to a condition in which the outcomes of the actions were sometimes absent. We predicted that we would observe larger RPs in the condition of constant than of inconstant outcomes.

## Methods

### Participants

Fourteen healthy participants (2 women, mean age = 23.9 years, *SD* = 1.4 years) took part in the experiment. All the participants were right-handed and reported normal or corrected-to-normal visual acuity. A power calculation using G*Power 3^13,14^ and an effect size estimated based on a previous RP study^12^ showed that this sample size was sufficient to produce a power larger than 0.95 (alpha = 0.05). Pornpattananangkul and Nusslock^12^ examined the effect of monetary reward on the RP, N2, and P3. They reported a large effect size (η^2^_p_ = 0.65) of reward on the RP. Based on this effect size, a sample size of 5 would be sufficient to provide a power of 0.95. However, because the effect of outcome reliability is probably weaker than that of monetary reward, we predicted a smaller effect size and chose a larger sample size of 14. The experiment was conducted according to the principles of the Helsinki Declaration and was approved by the ethics committee of the Faculty of Engineering at the University of Tokyo. All participants provided written informed consent prior to participation.

### Task and stimuli

**Figure 1:**
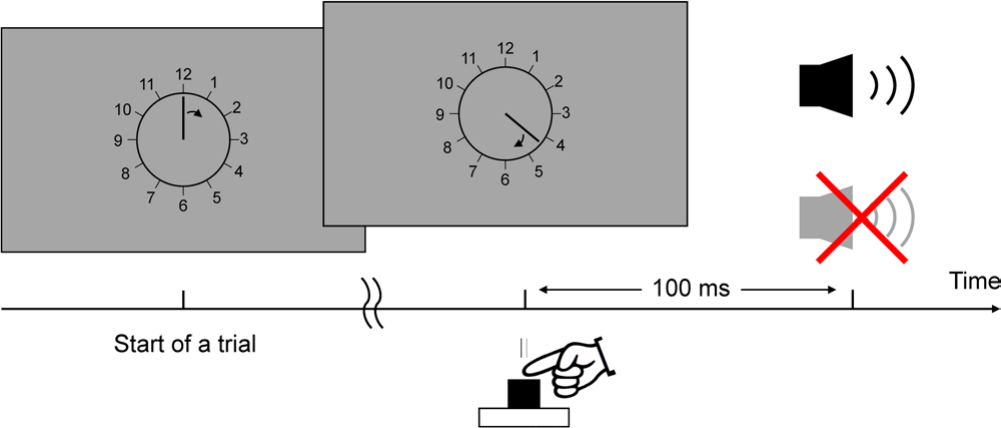
Timeline example for one trial.

We used a task modified from Libet *et al*.’s (1983). Figure 1 shows the timeline of an experimental trial. At the start of each trial, a clock face (visual angle = 4°) was shown on the screen and a clock hand pointed to 12′o clock. Participants were told to fix their eyes on the centre of the clock, avoiding any eye movements or eye blinks. The experimenter started the trial when the participant reported being ready. The clock hand rotated with a period of 2560 ms per cycle. Participants were instructed to wait for one cycle, and then press a button with their right index finger at a time of their choosing. Participants were told to press the button rapidly without hesitation upon deciding to act. The button (V-15-1A6, Omron, Japan) required a pressing force of 2.7 N. The clock hand kept on rotating for a random duration between 1.5–2.4 s. In the condition of constant outcome, a beep sound with a frequency of 500 Hz was always presented, via headphones, 100 ms after the button was pressed for 100 ms in each trial. In the condition of inconstant outcome, the beep sound was presented in only half the trials. Participants were told that sometimes the button works and triggers a beep sound, but sometimes it does not work and, therefore, does not trigger a sound. After the clock hand disappeared from the screen, the participants responded orally with a number to the question when they thought they had pressed the button.

We used an experimental design of one factor repeated measures. The within-subject factor was the constancy of consequence, which was blocked. In the block of constant outcome, participants pressed the button and then heard a beep sound in all 40 trials. In the block of inconstant outcome, participants pressed the button, but only heard the beep sound in 40 trials, and did not hear any sound in another 40 trials. In summary, the block of constant outcome consisted of 40 trials, and the block of inconstant outcome consisted of 80 trials. The order of the trials was randomized in each block, and the order of the blocks was counter-balanced between participants. In addition, the participant did not provide trial-by-trial report about their feeling of control over the beep sound (i.e. if they believed that they were always able to trigger the sound) because such subject report may heavily rely on reconstructive processes. In our task, the feedback of action consequence was extremely unambiguous (i.e. either present or absent), and would dominate the self-report. Instead, we confirmed with introspection that all the participants felt that there was a reliable association between their action and the beep sound in the constant condition, but were not sure whether or not their action would produce a consequence in the inconstant block. Importantly, we only used the trials in which the participants’ key presses produced beep sounds for the analysis of RP; thus, the trials for analyses had exactly the same physical feature (i.e. a keypressfollowed by a beep sound).

### Procedure

The task and EEG measurements were conducted in an electromagnetically shielded room. Participants sat 50 cm away from a 21-inch LCD monitor, wore an EEG electrode cap, and placed their right hand on the button remaining as still as possible during the task. After being informed about the task, participants practiced for 10 trials according to the type of the following block before each block. Participants took 5-min breaks between blocks and short breaks between trials, as needed. The experiment lasted an average of 2 hours.

### EEG measurement and preprocessing

EEG signals were recorded from Fp1, Fp2, F3, Fz, F4, FC5, FC1, FCz, FC2, FC6, T7, C3, C1, Cz, C2, C4, T8, CP5, CP1, CPz, CP2, Cp6, P3, Pz, P4, O1, and O2 according to the 10–20 system using g.LADYbird active ring electrodes and g.USBamp amplifiers (g.tec, Graz, Austria). Horizontal and vertical eye movements were recorded from the outer canthus of and below the right eye. An electromyogram (EMG), associated with the action of pressing the button, was recorded from a pair of the same type of electrodes, which were attached over the flexor carpi radialis muscle of the right arm^15^. The ground electrode was placed on AFz. All the EEG and EMG signals were referenced online against the right earlobe and were, then, re-referenced to an average of the left and right earlobe offline. A sampling frequency of 512 Hz was used.

The EMG signals were used to detect the onset of the movement of pressing the button. A high-pass filter of 20 Hz was applied to the EMG signals offline. The time window of 400-0 ms before the button was pressed (when the computer detected electric signals sent by the button) was used to detect the onset of the action of pressing the button. The onset of the action of pressing the button was defined as the first point that the EMG exceeded eight times the variance from the average between 500−400 ms prior to pressing the button.

EEG signals were pre-processed using the EEGLAB toolbox^16^ on MATLAB R2016a (MathWorks, Natick, MA). A notch filter of 50 Hz was applied before the rejection of eye artefacts. EEG signals were segmented into time-locked epochs ranging from 2500 ms before and 1000 ms after the onset of pressing the button^17^. Epochs containing large artefacts (±250 μV) were removed, and independent component analysis was used to remove eye movement artefacts. Epochs exceeding ± 100 μV at any channel were rejected. The valid trial number used for analyses was 34.4 and 35.9 on average (*SD*s* = *3.1 and 3.5) for the inconstant and constant conditions, respectively. A low-pass filter of 30 Hz was then applied. EEG signals were corrected using a 200-ms baseline before the time window of the readiness potential and averaged into ERPs for each condition at electrode Cz (i.e. the time window of baseline correction was [−2700–2500] ms).

## Results

### Behavioural results

Only the trials in which a tone was presented after the button press were used for further analyses. The 40 trials in which the tone was absent were designed to interrupt the constancy between action and feedback and were discarded from the analyses. Therefore, the trials for the analyses in the two experimental conditions (constant vs inconstant) had exactly the same physical features and number. The only difference between the two conditions was the reliability of the action consequence. In addition, the trials rejected in the EEG preprocessing were also discarded from the behavioural analyses.

The participants started pressing the button at 3.67 (*SD* = 0.42) and 3.74 (*SD* = 0.67) s, on average, after the clock hand started to rotate in the conditions of constant and inconstant outcome, respectively. There was no significant difference between the two conditions (*t*(13) = 0.84, *n.s*.). Furthermore, the accuracy in perception of the time of pressing the button (i.e. difference between the actual and reported time) did not differ between the two conditions, either (0.19 vs 0.35 s earlier than the actual time of pressing the button, *t*(13) = 0.97, *n.s*.). Finally, in order to examine whether the participants pressed the button with the same force between conditions, we calculated the maximum EMG for each trial and compared the averaged value between conditions. The maximum EMG did not differ between the conditions with constant and inconstant outcome (219 vs 216 μV, *SD*s = 87 and 86 μV, respectively, *t*(13) = 0.14, *n.s*.). In summary, the behavioural results showed that the physical aspects and the perception of the action did not differ between the two conditions.

### Readiness Potential

**Figure 2:**
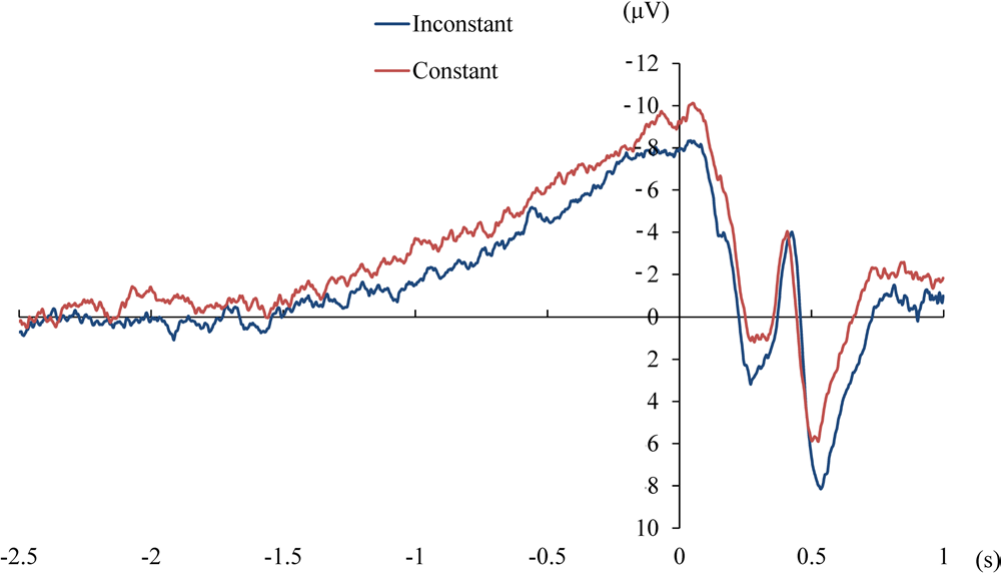
The mean readiness potential at Cz in the condition of constant and inconstant outcomes.

**Figure 3:**
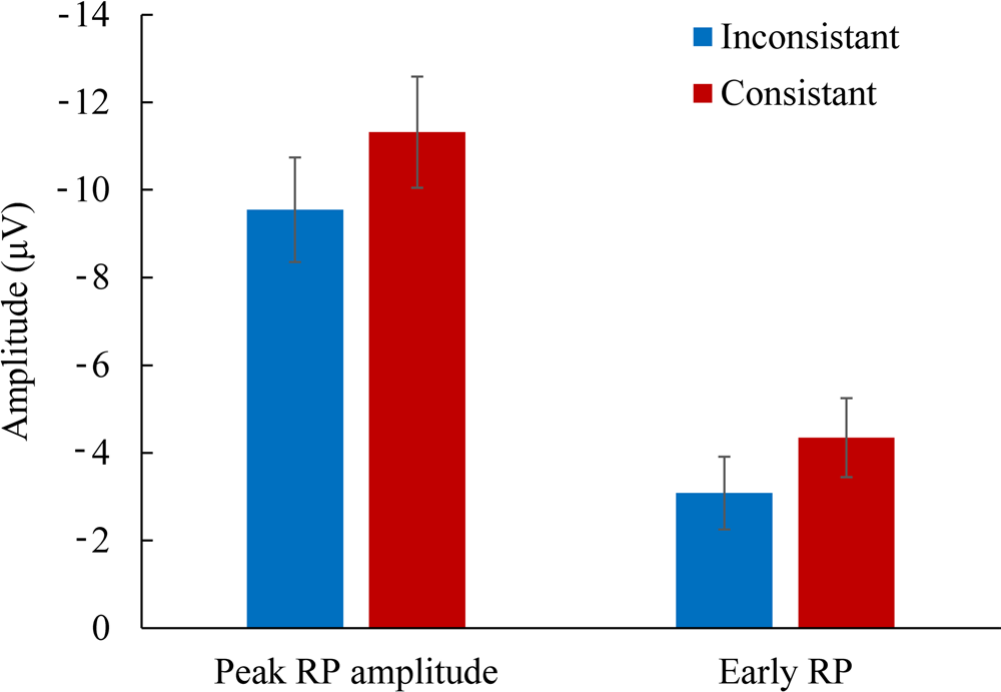
The average peak amplitude and early component of the readiness potential (RP) in each condition at Cz. Error bars represent standard errors.

**Figure 4:**
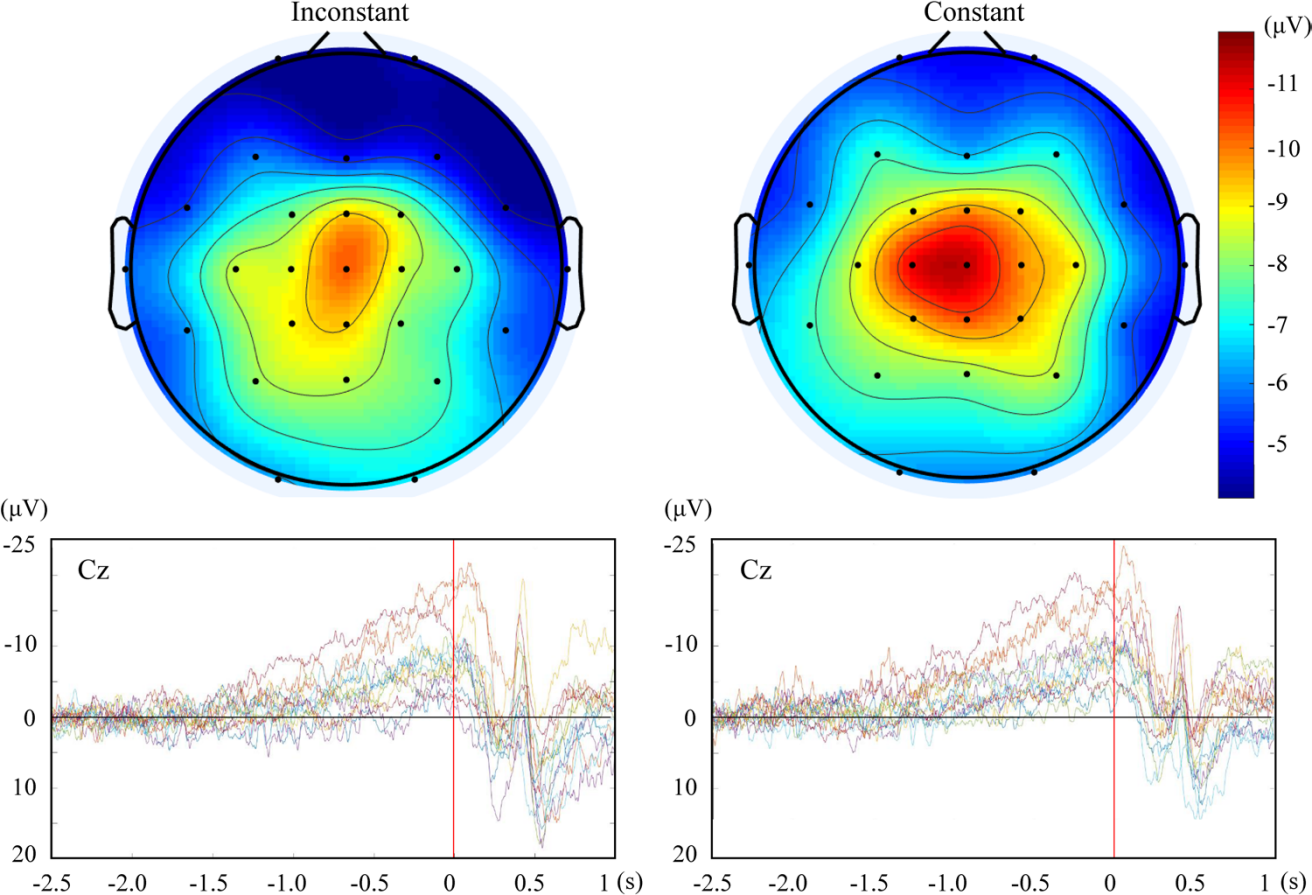
The topographies of peak amplitudes of the readiness potential (RP) (upper panel) and the individual RPs at Cz (lower panel) in the inconstant and constant conditions.

Figure 2 shows the mean RP time-locked to the onset of pressing the button at the electrode of Cz. Figure 3 shows the mean of the peak amplitudes and early components of the RP (see below) in each condition. Figure 4 shows the topographies of the peak RPs and individual RPs at Cz in the two experimental conditions.

The topographies showed that the RPs were localized to the central-parietal electrodes (with the maximum value at Cz, Fig. 4). The peak amplitudes of the RP were −9.5 (*SD* = 4.5) and −11.3 (*SD* = 4.8) μV in the conditions of inconstant and constant outcome at Cz, respectively. The RP in the condition of constant outcome was significantly larger than that in the condition of inconstant outcome (*t*(13) = 2.72, *p* = 0.017, Cohen’s d = 0.73). Further, we analysed the early RP by calculating the mean value of the potential in the time window of 1–0.5 s before the onset of the action^7,18^. The early RP does not contain the peak of RP and is considered to reflect the intention to move^7^. The early RP was larger in the condition of constant compared to inconstant outcome (−4.3 vs −3.1 μV, *SD* = 3.4 and 3.1 μV, respectively, *t*(13) = 2.19, *p* = 0.048, Cohen’s d = 0.58).

## Discussion

The present study examined the readiness potential (RP) preceding action in conditions where the participants received a reliable action outcome (i.e. an immediate beep sound) or not. In the condition of constant outcome, the participants were able to perceive the contingency between their action (i.e. pressing the button) and the beep sound, and a stronger RP was observed in both the early and late phase, compared to the condition in which the beep sound was sometimes absent after pressing the button. In contrast, the EMG measurements and the behavioural results showed that there was no significant difference in the physical aspects or the perception of the action between the two conditions. Therefore, our results showed that the reliability of action consequence did not affect the action’s execution but influenced neural activities prior to its performance.

In the present study, because the participants knew that they had to press a key in all trials, there was no freedom in the intention to act. However, in the inconstant condition, because the beep sound was absent in half of the trials, the participants probably received a trial in which there was no outcome after their action from the very beginning of the block. Such outcome-absent trials impaired the action-outcome contingency and probably lowered the anticipation of the outcome, although the participants were not instructed to pay attention to the outcomes. Our results regarding the RP showed that the perceived action-outcome contingency enhanced the neural activities preceding the action, even when it was task irrelevant. Our results support the view that a reliable action feedback is rewarding to the organism^2,19^, consequently affecting action preparation.

Although the present literature presumes that the RP reflects planning and preparation for action, recent research provided alternative accounts for the nature of the RP. Schurger *et al*. suggested that the RP is the result of the accumulation of artefacts before an action, and such artefacts (i.e. noises) are imperative to causing an action when they reach a threshold^20^. In other words, the RP may not be a valid event-related potential. Furthermore, a recent study suggested that a systematic decrease of cross-trial variability in the EEG before an action also reflected the intention to act, as does the mean RP^21^. Taken together, as the RP is usually absent in each single trial but present in the accumulation of neural signals before an action, it remains controversial whether and how the mean RP is directly associated with the intention or motor preparation of each single action. Nevertheless, our results revealed that the anticipation or prediction of an action consequence indeed greatly influenced the neural activities prior to the action, showing that the neural activities preceding an action are associated with higher-level processes, rather than random neural artefacts.

In conclusion, the present study showed that reliable action consequences resulted in larger RPs in both the early and late phases, compared to the condition in which the presence of the consequence was unreliable. The results indicated that the contingency between action and outcomes probably rewards the action and has great influence over the neural activities before an action.

## References

[1] Rovee CK, Rovee DT (1969). Conjugate reinforcement of infant exploratory behavior. J. Exp. Child Psychol..

[2] Karsh N, Eitam B (2015). I control therefore I do: Judgments of agency influence action selection. Cognition.

[3] Shibasaki H, Hallett M (2006). What is the Bereitschaftspotential?. Clin. Neurophysiol..

[4] Deecke L, Scheid P, Kornhuber HH (1969). Distribution of readiness potential, pre-motion positivity, and motor potential of the human cerebral cortex preceding voluntary finger movements. Exp. Brain Res..

[5] Libet B, Gleason Ca, Wright EW, Pearl DK (1983). Time of conscious intention to act in relation to onset of cerebral activity (readiness-potential). Brain.

[6] Libet B (1999). Do we have free will?. J. Conscious. Stud..

[7] Haggard P, Eimer M (1999). On the relation between brain potentials and the awareness of voluntary movements. Exp. Brain Res..

[8] Haggard P (2017). Sense of agency in the human brain. Nat. Rev. Neurosci..

[9] Slobounov S, Hallett M, Newell KM (2015). Perceived effort in force production as reflected in motor-related cortical potentials. Clin. Neurophysiol..

[10] Masaki H, Takasawa N, Yamazaki K (1998). Enhanced negative slope of the readiness potential preceding a target force production task. Electroencephalogr. Clin. Neurophysiol. Potentials Sect..

[11] Benecke R, Dick JPR, Rothwell JC, Day BL, Marsden CD (1985). Increase of the Bereitschaftspotential in simultaneous and sequential movements. Neurosci. Lett..

[12] Pornpattananangkul N, Nusslock R (2015). Motivated to win: Relationship between anticipatory and outcome reward-related neural activity. Brain Cogn..

[13] Faul F, Erdfelder E, Buchner A, Lang A-G (2009). Statistical power analyses using G*Power 3.1: tests for correlation and regression analyses. Behav. Res. Methods.

[14] Faul F, Erdfelder E, Lang A-G, Buchner A (2007). G*Power 3: A flexible statistical power analysis program for the social, behavioral, and biomedical sciences. Behav. Res. Methods.

[15] Alexander P (2016). Readiness potentials driven by non-motoric processes. Conscious. Cogn..

[16] Delorme A, Makeig S (2004). EEGLAB: An open sorce toolbox for analysis of single-trial EEG dynamics including independent component anlaysis. J. Neurosci. Methods.

[17] Jo H-G, Wittmann M, Hinterberger T, Schmidt S (2014). The readiness potential reflects intentional binding. Front. Hum. Neurosci..

[18] Schlegel A (2013). Barking up the wrong free: Readiness potentials reflect processes independent of conscious will. Exp. Brain Res..

[19] Karsh N, Eitam B, Mark I, Higgins ET (2016). Bootstrapping agency: How control-relevant information affects motivation. J. Exp. Psychol. Gen..

[20] Schurger a, Sitt JD, Dehaene S (2012). An accumulator model for spontaneous neural activity prior to self-initiated movement. Proc. Natl. Acad. Sci..

[21] Khalighinejad N, Schurger A, Desantis A, Zmigrod L, Haggard P (2018). Precursor processes of human self-initiated action. Neuroimage.

